# Equivocal and definite interstitial lung abnormalities as risk factors for trastuzumab deruxtecan-related interstitial lung disease in breast cancer

**DOI:** 10.1093/oncolo/oyag420

**Published:** 2026-10-19

**Authors:** Tomoaki Nakamura, Naoki Murata, Yukiko Hibino, Clara So, Atsushi Kitamura, Yutaka Tomishima, Torahiko Jinta

**Affiliations:** Department of Pulmonary Medicine, Thoracic Center, St. Luke’s International Hospital, 9-1 Akashi-cho, Chuo-ku, Tokyo 104-8560, Japan; Department of Thoracic Oncology, National Cancer Center Hospital, 5-1-1 Tsukiji, Chuo-ku, Tokyo 104-0045, Japan; Department of Pulmonary Medicine, Thoracic Center, St. Luke’s International Hospital, 9-1 Akashi-cho, Chuo-ku, Tokyo 104-8560, Japan; Department of Medical Oncology, St. Luke’s International Hospital, 9-1 Akashi-cho, Chuo-ku, Tokyo 104-8560, Japan; Division of Pulmonary, Critical Care, Allergy and Sleep, Department of Medicine, University of California, San Francisco, San Francisco, CA 94143, United States; Department of Pulmonary Medicine, Thoracic Center, St. Luke’s International Hospital, 9-1 Akashi-cho, Chuo-ku, Tokyo 104-8560, Japan; Department of Pulmonary Medicine, Thoracic Center, St. Luke’s International Hospital, 9-1 Akashi-cho, Chuo-ku, Tokyo 104-8560, Japan; Department of Pulmonary Medicine, Thoracic Center, St. Luke’s International Hospital, 9-1 Akashi-cho, Chuo-ku, Tokyo 104-8560, Japan

**Keywords:** trastuzumab deruxtecan, interstitial lung abnormalities, interstitial lung disease, breast cancer

## Abstract

**Background:**

Trastuzumab deruxtecan (T-DXd) is effective for advanced breast cancer but is associated with interstitial lung disease (ILD). The clinical relevance of baseline interstitial lung abnormalities (ILAs), including subtle equivocal findings, remains unclear.

**Methods:**

This retrospective cohort study included consecutive patients with advanced or recurrent breast cancer who initiated T-DXd at a tertiary hospital in Japan between June 2020 and May 2025. Baseline CT before T-DXd initiation was reviewed for ILAs and classified as no ILA, equivocal ILA, or definite ILA. The cumulative incidence of T-DXd-related ILD was estimated using a competing-risk approach.

**Results:**

Among 156 patients, baseline ILAs were identified in 50 patients: 21 with equivocal ILA and 29 with definite ILA. T-DXd-related ILD occurred in 31 patients (20%), including 19 of 50 patients (38%) in the ILA group and 12 of 106 patients (11%) in the non-ILA group. Baseline ILA was associated with a higher cumulative incidence of T-DXd-related ILD (subdistribution hazard ratio, 3.56; 95% CI, 1.63-7.78; *P* = .001). No significant difference in T-DXd-related ILD risk was detected between the equivocal and definite ILA groups. This association remained consistent in the <1.5-mm CT subgroup. No significant differences in progression-free or overall survival were observed according to baseline ILA status. Among patients with baseline ILA, 33 of 50 had ILAs first detected during prior systemic anticancer therapy.

**Conclusions:**

Baseline ILAs, including equivocal findings, were associated with an increased risk of T-DXd-related ILD. ILAs were frequently first detected during prior systemic anticancer therapy before T-DXd initiation.

## Graphical Abstract

**Graphical Abstract:**
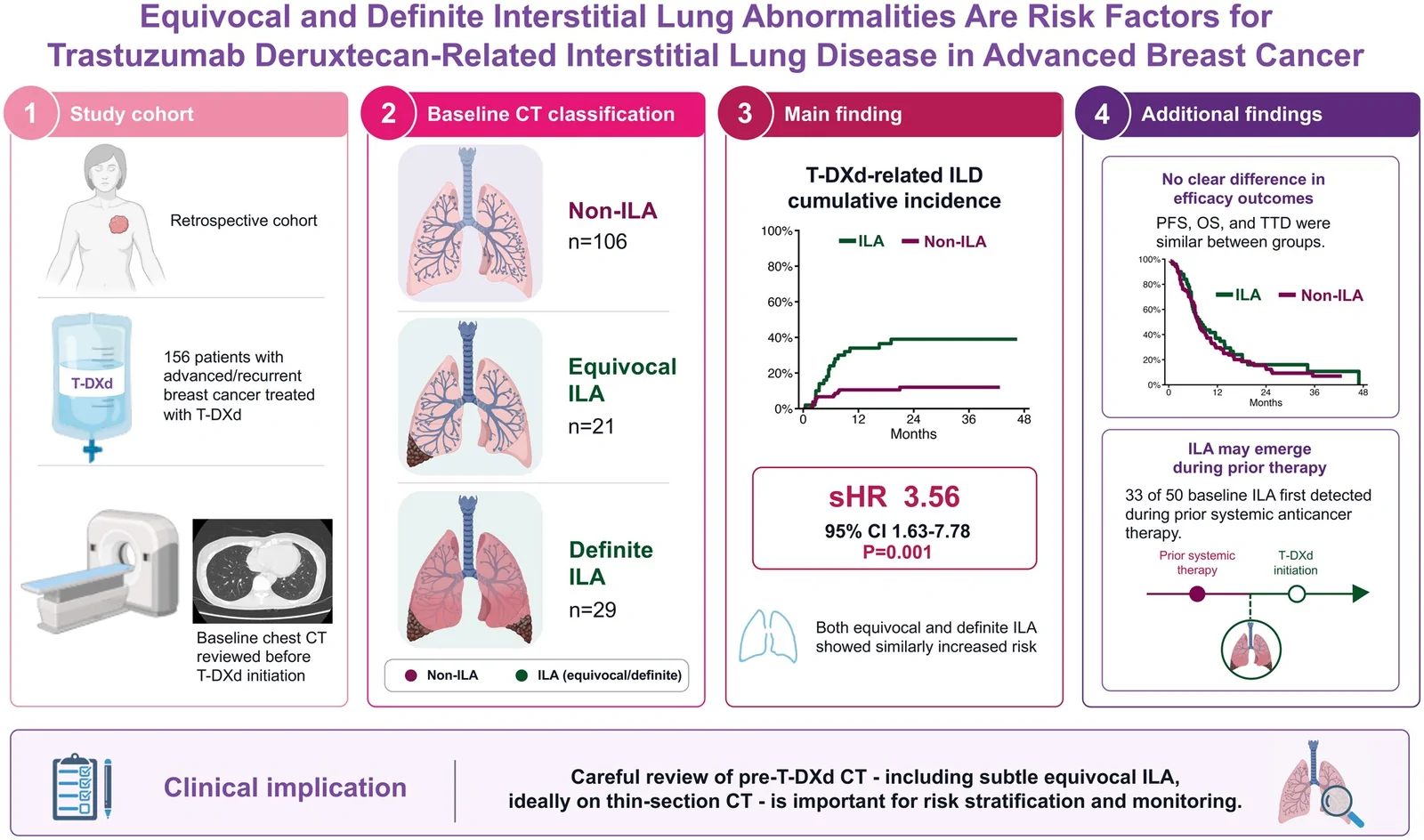

Implications for practice Baseline interstitial lung abnormalities, including subtle equivocal findings, may identify patients with advanced or recurrent breast cancer who are at increased risk of trastuzumab deruxtecan-related interstitial lung disease. Careful review of the most recent pretreatment chest CT, preferably using thin-section images when available, may help clinicians refine risk assessment before treatment initiation. These findings support closer radiologic and clinical monitoring in patients with baseline interstitial lung abnormalities, while emphasizing that such findings should inform individualized management rather than automatically preclude trastuzumab deruxtecan therapy.

## Introduction

Trastuzumab deruxtecan (T-DXd) has demonstrated substantial clinical benefit in patients with advanced or recurrent breast cancer across a broad range of HER2 expression levels.^1–3^ However, T-DXd-related interstitial lung disease (ILD) remains a clinically important adverse event because it can be severe and occasionally fatal.^4^ Thus, identifying baseline radiological factors associated with T-DXd-related ILD is essential for safe treatment selection and monitoring.

Pooled analyses of T-DXd monotherapy trials have reported several risk factors for T-DXd-related ILD, including older age, Japanese origin, preexisting lung comorbidities, renal dysfunction, longer time since initial diagnosis, higher dose, and lower baseline oxygen saturation.^4^ Nevertheless, preexisting lung comorbidities encompass diverse conditions, and the clinical impact of subtle baseline CT abnormalities has not been fully clarified.

Interstitial lung abnormalities (ILAs) are CT findings suggestive of early or subclinical ILD.^5^ In other anticancer treatment settings, preexisting ILD and ILAs have been associated with an increased risk of treatment-related ILD.^6^^,^^7^ However, the clinical significance of baseline ILAs in patients receiving T-DXd for breast cancer remains unclear. Moreover, because the detection of subtle ILAs may be affected by CT acquisition parameters, including slice thickness, careful assessment of baseline CT images is important.^5^^,^^8^

In this study, we assessed ILAs on baseline CT before T-DXd initiation and examined their association with the development of T-DXd-related ILD in patients with advanced or recurrent breast cancer.

## Materials and methods

### Study design and patients

This retrospective cohort study was conducted at a tertiary hospital in Japan. We reviewed consecutive patients with advanced or recurrent breast cancer who initiated T-DXd between June 2020 and May 2025. Patients were eligible if they received at least one dose of T-DXd during the study period. Patients without an available baseline chest CT before T-DXd initiation, including those with PET-CT alone, were excluded. Baseline CT was defined as the most recent chest CT performed within 30 days before T-DXd initiation. The data cutoff date was April 30, 2026.

### Data collection

Clinical data were retrospectively collected from medical records. Baseline variables at T-DXd initiation included age, sex, smoking history, Eastern Cooperative Oncology Group performance status, disease stage, intrinsic subtype, liver metastasis, brain metastasis, prior breast or chest wall radiotherapy, history of systemic anticancer therapy for advanced or recurrent breast cancer, and baseline CT slice thickness. Systemic anticancer therapy for advanced or recurrent breast cancer included endocrine therapy, chemotherapy, anti-HER2 therapy, CDK4/6 inhibitors, and other targeted agents administered before T-DXd initiation. Treatments given before recurrence, including neoadjuvant and adjuvant therapies, were not included. Baseline CT slice thickness was recorded and categorized as <1.5 mm or ≥1.5 mm because thin-section CT has been reported to improve the detection of subtle ILAs. Treatment and follow-up data included the date of T-DXd initiation, the date of T-DXd discontinuation, the occurrence and date of T-DXd-related ILD, ILD grade, disease progression, death, and the date of last follow-up.

#### Assessment of ILAs

Baseline CT images were evaluated for ILAs before T-DXd initiation with reference to the 2025 American Thoracic Society clinical statement.^8^ Because this study aimed to evaluate subtle abnormalities that do not meet the formal ATS definition of ILA, we used a study-specific 3-level classification adapted from the COPDGene framework.^9^ Clinical records and baseline CT images were also reviewed to identify clinically diagnosed ILD before T-DXd initiation. CT findings potentially compatible with ILD, including non-dependent ground-glass opacity, reticular abnormalities, traction bronchiectasis, architectural distortion, non-emphysematous cysts, and honeycombing, were evaluated. Findings unlikely to represent ILA, including dependent atelectasis, focal paraspinal fibrosis, pulmonary edema, infection, radiation-related change confined to the prior radiation field, lymphangitic carcinomatosis, and tumor-related abnormalities, were excluded from the ILA assessment whenever possible.

Each lung was divided into 3 zones according to the levels of the inferior aortic arch and right inferior pulmonary vein. ILA status was classified as no ILA, equivocal ILA, or definite ILA. Equivocal ILA was defined as either a subtle interstitial abnormality involving <5% of any lung zone, such as limited non-dependent ground-glass opacity or reticular abnormality, or a unilateral interstitial abnormality regardless of its extent. Definite ILA was defined as bilateral interstitial abnormalities involving ≥5% of any lung zone. For descriptive characterization, definite ILAs were further categorized as non-subpleural, subpleural non-fibrotic, or subpleural fibrotic. Patients with equivocal or definite ILA were classified into the ILA group, whereas those without ILA were classified into the non-ILA group. Thus, this 3-level classification was study-specific and was distinct from the formal ATS ILA framework.

Baseline CT images were independently reviewed by 2 board-certified pulmonologists experienced in drug-related ILD and chest imaging. Disagreements were resolved by consensus. Baseline ILA classification, including consensus adjudication, was completed before the review of post-T-DXd CT images for ILD assessment. Interobserver agreement for the 3-level ILA classification was evaluated using the independent assessments before consensus adjudication. Available CT images before the baseline CT were reviewed to determine the timing of first ILA detection. Patients were classified as having pretreatment ILA if ILA was already present before the initiation of systemic anticancer therapy for advanced or recurrent breast cancer, and as having treatment-course ILA if ILA was first detected during systemic anticancer treatment before T-DXd initiation. For the timing analysis, the duration of prior systemic anticancer therapy was defined as the interval from initiation of systemic anticancer therapy for advanced or recurrent breast cancer to T-DXd initiation.

### Assessment of T-DXd-related ILD

After baseline ILA assessment, CT images obtained after T-DXd initiation, including after the last dose, were reviewed chronologically for ILD alongside relevant medical records. Suspected ILD events were evaluated based on clinical symptoms, the temporal relationship with T-DXd exposure, chest imaging findings, laboratory and microbiological data when available, the treating physician’s clinical assessment, and the exclusion of alternative diagnoses, including infection, pulmonary edema, heart failure, radiation pneumonitis, pulmonary embolism, and tumor progression. In patients with baseline ILA, new bilateral, diffuse, relatively homogeneous opacities favored drug-related ILD over progression of preexisting ILA. Radiation-related lung injury was differentiated by its relationship to the prior radiation field. Treating physicians initially assessed suspected ILD; 2 pulmonologists independently re-adjudicated cases for this study, resolving disagreements by consensus.

ILD severity was graded according to the Common Terminology Criteria for Adverse Events version 5.0. Radiological patterns of T-DXd-related ILD were classified as hypersensitivity pneumonitis, organizing pneumonia, or nonspecific interstitial pneumonia. The date of ILD onset was defined as the date when new radiological abnormalities or clinical symptoms leading to the diagnosis of ILD were first documented.

### Statistical analysis

Continuous variables are presented as medians and interquartile ranges, and categorical variables as numbers and percentages. Baseline characteristics were summarized descriptively and were not subjected to formal hypothesis testing. Exploratory comparisons of characteristics according to the timing of ILA detection and characteristics among patients who developed T-DXd-related ILD were performed using Fisher’s exact test for categorical variables and the Wilcoxon rank-sum test for continuous variables. Interobserver agreement for the 3-level ILA classification was assessed using linearly weighted Cohen’s κ, with 95% CIs estimated using percentile bootstrap resampling (20 000 resamples). CT imaging frequency was assessed as days per chest CT scan, calculated by dividing the interval from T-DXd initiation to the earliest of ILD diagnosis, 21 days after the last T-DXd administration, death, or last follow-up by the number of chest CT scans obtained during that interval.

The primary outcome was the cumulative incidence of T-DXd-related ILD. Secondary outcomes included severe T-DXd-related ILD, progression-free survival (PFS), overall survival (OS), and time to treatment discontinuation (TTD). Severe ILD was defined as grade ≥3 ILD. PFS was defined as the time from T-DXd initiation to disease progression or death from any cause. OS was defined as the time from T-DXd initiation to death from any cause. TTD was defined as the time from T-DXd initiation to the last T-DXd administration, with ongoing treatment censored at the last administration date.

The cumulative incidence of T-DXd-related ILD was estimated using a competing risk approach. In the primary analysis, death without ILD and discontinuation of T-DXd before ILD onset were treated as competing events. As a sensitivity analysis, T-DXd discontinuation before ILD was treated as censoring, with death without ILD as the only competing event. Cumulative incidence estimates and corresponding 95% CIs were calculated at 6 and 12 months. Differences in cumulative incidence between groups were evaluated using Gray’s test. For comparisons among ILA categories, pairwise Gray tests were performed, and *P* values were adjusted using the Holm method.

Fine–Gray subdistribution hazard models were used to estimate the association between baseline ILA and the cumulative incidence of T-DXd-related ILD. Cause-specific Cox models censored patients at competing events; univariable and multivariable models were fitted. Multivariable models adjusted for age, given its association with ILA prevalence, and prior breast/chest wall radiotherapy, a potential pulmonary risk factor. A sensitivity multivariable model additionally included smoking history. A separate sensitivity analysis was performed in patients whose baseline CT had a slice thickness <1.5 mm.

PFS and OS were estimated using the Kaplan–Meier method and compared using the log-rank test. Median follow-up was estimated using the reverse Kaplan–Meier method. Cox proportional hazards models were used to estimate hazard ratios and 95% CIs. Multivariable Cox models for PFS and OS were adjusted for age, ECOG performance status, disease stage, liver metastasis, and brain metastasis, which were selected as clinically relevant prognostic factors in advanced breast cancer.

All statistical tests were 2-sided, and *P* values <.05 were considered statistically significant. Statistical analyses were performed using R software version 4.5.2.

## Results

### Patient flow and baseline characteristics

A total of 157 patients with advanced or recurrent breast cancer initiated T-DXd during the study period. After excluding one patient with PET-CT only and no available baseline chest CT, 156 patients were included in the analysis (Figure S1). Baseline ILA was identified in 50 patients (21 equivocal, 29 definite), versus 106 without ILA (Table 1). No patient had clinically diagnosed ILD at baseline before T-DXd initiation. All received T-DXd monotherapy; 90% started at 5.4 mg/kg (Table 1). Patients in the ILA group tended to be older, whereas other baseline characteristics and CT slice thickness distribution were generally similar between groups. Baseline CT with a slice thickness <1.5 mm was available in 40 patients (80%) in the ILA group and 83 patients (78%) in the non-ILA group (Table S1). Interobserver agreement was substantial (weighted κ, 0.721; 95% CI, 0.646-0.793; exact agreement, 81.4%; Table S2). Median days per chest CT scan were 85.4 (IQR, 70.0-105.0) for ILA versus 101.8 (IQR, 77.0-118.1) for non-ILA (*P* = .057).

**Table 1.** Clinical characteristics of patients.

Characteristic	Total	ILA	Non-ILA
	*N* = 156	N1 = 50	N2 = 106
**Age, years**	56.0 (49.0, 63.0)	60.5 (54.0, 66.0)	54.0 (46.0, 61.0)
**Sex**			
** Female**	156 (100%)	50 (100%)	106 (100%)
**Ethnic/geographic background**			
** Japanese**	153 (98%)	48 (96%)	105 (99%)
** Non-Japanese^a^**	3 (1.9%)	2 (4.0%)	1 (0.9%)
**Smoking history**	19 (12%)	7 (14%)	12 (11%)
**Performance status**			
** 0-1**	137 (88%)	45 (90%)	92 (87%)
** 2-4**	19 (12%)	5 (10%)	14 (13%)
**Stage**			
** Recurrent**	106 (68%)	33 (66%)	73 (69%)
** Stage III-IV**	50 (32%)	17 (34%)	33 (31%)
**Intrinsic subtype**			
** Luminal**	125 (80%)	39 (78%)	86 (81%)
** HER2**	26 (17%)	8 (16%)	18 (17%)
** TNBC**	5 (3.2%)	3 (6.0%)	2 (1.9%)
**Liver metastasis**	84 (54%)	31 (62%)	53 (50%)
**Brain metastasis**	18 (12%)	5 (10%)	13 (12%)
**Creatinine clearance, mL/min^b^**	86.6 (70.8, 102.1)	86.1 (75.0, 101.8)	87.1 (67.3, 102.3)
**Prior treatment**			
** Breast/chest wall RT**	62 (40%)	19 (38%)	43 (41%)
** ICI**	7 (4.5%)	2 (4.0%)	5 (4.7%)
** T-DM1**	41 (26%)	14 (28%)	27 (25%)
**Number of prior lines of therapy for advanced/recurrent disease**	3.0 (2.0, 5.0)	2.0 (2.0, 5.0)	3.0 (2.0, 5.0)
**Slice thickness of baseline CT, mm**			
** 0.5**	25 (16%)	5 (10%)	20 (19%)
** 0.625**	79 (51%)	30 (60%)	49 (46%)
** 1**	3 (1.9%)	2 (4.0%)	1 (0.9%)
** 1.25**	16 (10%)	3 (6.0%)	13 (12%)
** 2.5**	33 (21%)	10 (20%)	23 (22%)
**Baseline CT slice thickness <1.5 mm**	123 (79%)	40 (80%)	83 (78%)
**ILA type on baseline CT**			
** Equivocal ILA**	—	21 (42%)	—
** Definite ILA**	—	29 (58%)	—
**Initial T-DXd dose, mg/kg**			
** 5.4**	140 (90%)	43 (86%)	97 (92%)
** 4.4**	14 (9.0%)	7 (14%)	7 (6.6%)
** 3.2**	2 (1.3%)	0 (0%)	2 (1.9%)

Continuous variables are expressed as medians (interquartile ranges), and categorical variables are expressed as *n* (%).

Abbreviations: ECOG PS, Eastern Cooperative Oncology Group performance status; ICI, immune checkpoint inhibitor; ILA, interstitial lung abnormality; RT, radiotherapy; T-DM1, trastuzumab emtansine; T-DXd, trastuzumab deruxtecan; TNBC, triple-negative breast cancer.

a The 3 patients of non-Japanese background were Chinese, Malaysian, and Filipino (*n* = 1 each).

b Creatinine clearance was estimated using the Cockcroft–Gault equation.

### T-DXd-related ILD according to baseline ILA status

T-DXd-related ILD occurred in 31 patients (20%), including 19 of 50 patients (38%) in the ILA group and 12 of 106 patients (11%) in the non-ILA group. In the primary competing-risk analysis, 25 of 50 patients in the ILA group and 76 of 106 patients in the non-ILA group were classified as competing events due to T-DXd discontinuation without a prior ILD event. No patients were classified as competing events because of death without ILD. The cumulative incidence of T-DXd-related ILD was significantly higher in the ILA group than in the non-ILA group (Figure 1A). In the multivariable Fine–Gray model adjusted for age and prior breast/chest wall radiotherapy, baseline ILA was independently associated with a higher cumulative incidence of T-DXd-related ILD (subdistribution hazard ratio [sHR], 3.56; 95% CI, 1.63-7.78; *P* = .001; Table S3). In a sensitivity analysis using a multivariable model that additionally included smoking history, the association remained similar (sHR, 3.53; 95% CI, 1.62-7.70; *P* = .002; Table S3). Similar findings were observed in cause-specific Cox analyses (Table S3). In a separate sensitivity analysis treating T-DXd discontinuation as censoring and death without ILD as the only competing event, the association remained similar in the multivariable model (sHR, 3.18; 95% CI, 1.47-6.84; *P* = .003).

**Figure 1.**
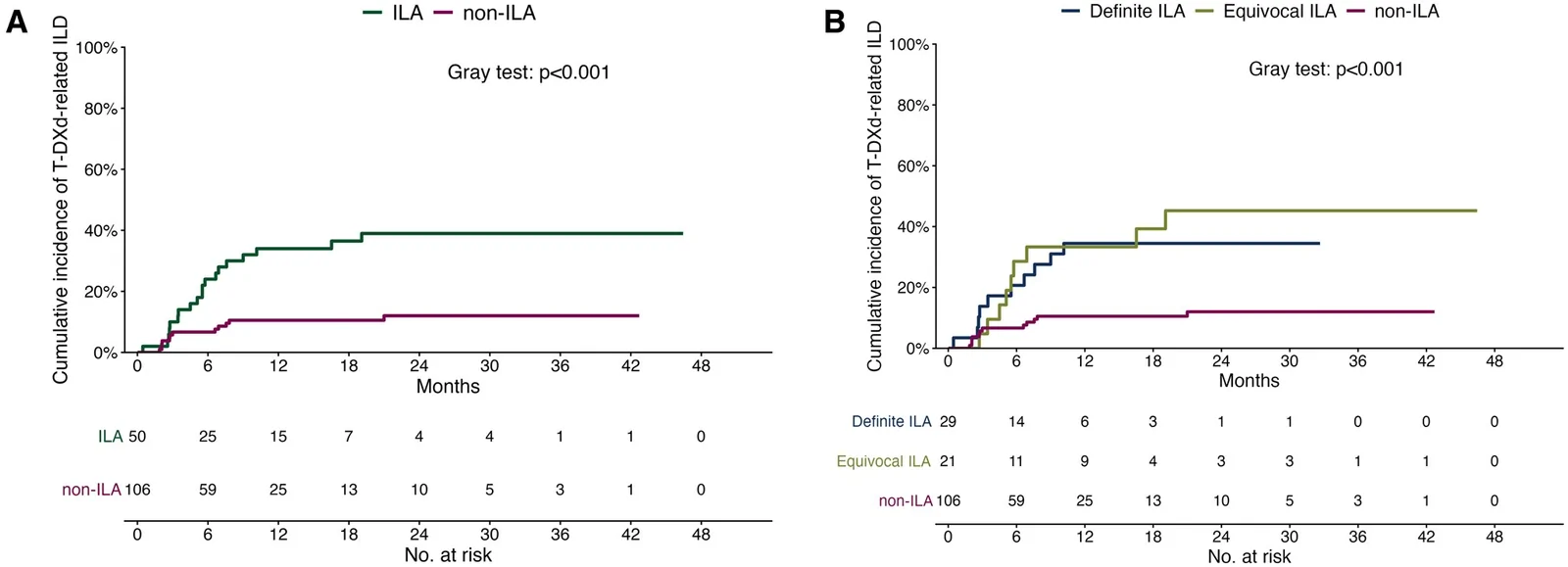
Cumulative incidence of T-DXd-related ILD according to baseline ILA status. (A) Presence or absence of baseline ILA. (B) ILA classification. Between-group differences were assessed using Gray’s test. ILD, interstitial lung disease; ILA, interstitial lung abnormality; T-DXd, trastuzumab deruxtecan.

When patients were stratified by ILA classification, T-DXd-related ILD occurred in 12 of 106 patients with non-ILA, 9 of 21 patients with equivocal ILA, and 10 of 29 patients with definite ILA. The cumulative incidence of T-DXd-related ILD was higher in both the definite and equivocal ILA groups than in the non-ILA group (Figure 1B). At 6 months, the cumulative incidences were 6.7% (95% CI, 1.9-11.5), 28.6% (95% CI, 8.6-48.5), and 20.7% (95% CI, 5.6-35.8), respectively. At 12 months, the corresponding incidences were 10.5% (95% CI, 4.6-16.5), 33.3% (95% CI, 12.5-54.2), and 34.5% (95% CI, 16.5-52.4), respectively. In pairwise comparisons, no significant difference was detected between the definite and equivocal ILA groups (Holm-adjusted *P* = .638; Table S4). Among the 29 patients with definite ILA, 7 had non-subpleural ILA and 22 had subpleural non-fibrotic ILA; no patients had subpleural fibrotic ILA. T-DXd-related ILD occurred in 5 of 7 patients (71.4%) with non-subpleural ILA and 5 of 22 patients (22.7%) with subpleural non-fibrotic ILA (Table S5).

To address the potential influence of CT slice thickness on ILA detection, we performed a sensitivity analysis restricted to patients whose baseline CT had a slice thickness <1.5 mm. In this subgroup, baseline ILA remained associated with T-DXd-related ILD in multivariable Fine–Gray and cause-specific Cox analyses adjusted for age and prior breast/chest wall radiotherapy (Figure S2A and Table S6). When patients were stratified by ILA classification, T-DXd-related ILD occurred in 9 of 83 patients with non-ILA, 8 of 15 patients with equivocal ILA, and 10 of 25 patients with definite ILA. At 6 months, the cumulative incidences were 6.1% (95% CI, 0.9-11.3), 33.3% (95% CI, 8.3-58.3), and 24.0% (95% CI, 6.8-41.2), respectively. At 12 months, the corresponding incidences were 9.8% (95% CI, 3.3-16.3), 40.0% (95% CI, 13.9-66.1), and 40.0% (95% CI, 20.0-60.0), respectively. In pairwise comparisons, no significant difference was detected between definite and equivocal ILA groups (Holm-adjusted *P* = .537; Figure S2B and Table S4).

### Characteristics of T-DXd-related ILD

Among the 31 patients who developed T-DXd-related ILD, the median cycle number at onset was 7.5 cycles (IQR, 4.0-11.0), and the median time to onset was 5.1 months (IQR, 2.7-7.6) (Table 2). Of the 31 T-DXd-related ILD events, 11 (35%) were diagnosed after the last T-DXd administration. Most events were grade 1 or 2; grade ≥3 ILD occurred in one patient (3.2%), and no grade 4 or 5 ILD occurred. Twenty-five patients (81%) were managed with T-DXd discontinuation alone, whereas 6 patients (19%) received corticosteroid therapy. Rechallenge after T-DXd-related ILD was performed in 8 patients (26%), all of whom had experienced grade 1 ILD. No recurrent T-DXd-related ILD was observed after rechallenge. The distribution of radiological patterns was not significantly different between the ILA and non-ILA groups.

**Table 2.** Characteristics among patients with T-DXd-related ILD.

Characteristic	Overall	ILA	Non-ILA	*P*-value
	*N* = 31	N1 = 19	N2 = 12	
**Cycle number at T-DXd-related ILD onset**	7.5 (4.0, 11.0)	8.5 (4.0, 11.0)	5.0 (3.0, 10.5)	.307
**Time to T-DXd-related ILD onset, months**	5.1 (2.7, 7.6)	5.5 (2.8, 7.6)	2.9 (2.1, 7.2)	.351
**T-DXd-related ILD grade**				>.999
** 1**	23 (74%)	14 (74%)	9 (75%)	
** 2**	7 (23%)	4 (21%)	3 (25%)	
** 3**	1 (3.2%)	1 (5.3%)	0 (0%)	
**ILD management**				>.999
** Discontinuation alone**	25 (81%)	15 (79%)	10 (83%)	
** Corticosteroid therapy**	6 (19%)	4 (21%)	2 (17%)	
**Radiological pattern of T-DXd-related ILD**				.389
** HP**	8 (26%)	6 (32%)	2 (17%)	
** OP**	21 (68%)	11 (58%)	10 (83%)	
** NSIP**	2 (6.5%)	2 (11%)	0 (0%)	
**Rechallenge after T-DXd-related ILD**	8 (26%)	5 (26%)	3 (25%)	>.999

Continuous variables are expressed as medians (interquartile ranges), and categorical data are expressed as *n* (%).

Abbreviations: ILA, interstitial lung abnormality; ILD, interstitial lung disease; HP, hypersensitivity pneumonitis; NSIP, nonspecific interstitial pneumonia; OP, organizing pneumonia; T-DXd, trastuzumab deruxtecan.

### Survival outcomes

The median follow-up for the overall cohort was 27.5 months (95% CI, 21.8-32.9). PFS and OS did not differ significantly according to baseline ILA status (Figure 2). The median PFS was 7.6 months in the ILA group and 7.4 months in the non-ILA group (hazard ratio [HR], 0.88; 95% CI, 0.61-1.28; *P* = .511). The median OS was 23.5 months (95% CI, 16.3-not estimable) in the ILA group and 23.1 months (95% CI, 21.4-36.1) in the non-ILA group (HR, 0.94; 95% CI, 0.59-1.52; *P* = .810). In multivariable Cox analyses, baseline ILA was not significantly associated with PFS or OS (Tables S7 and S8). TTD did not differ significantly between the ILA and non-ILA groups, with median values of 6.4 and 6.7 months, respectively (HR, 0.97; 95% CI, 0.67-1.41; *P* = .882).

**Figure 2.**
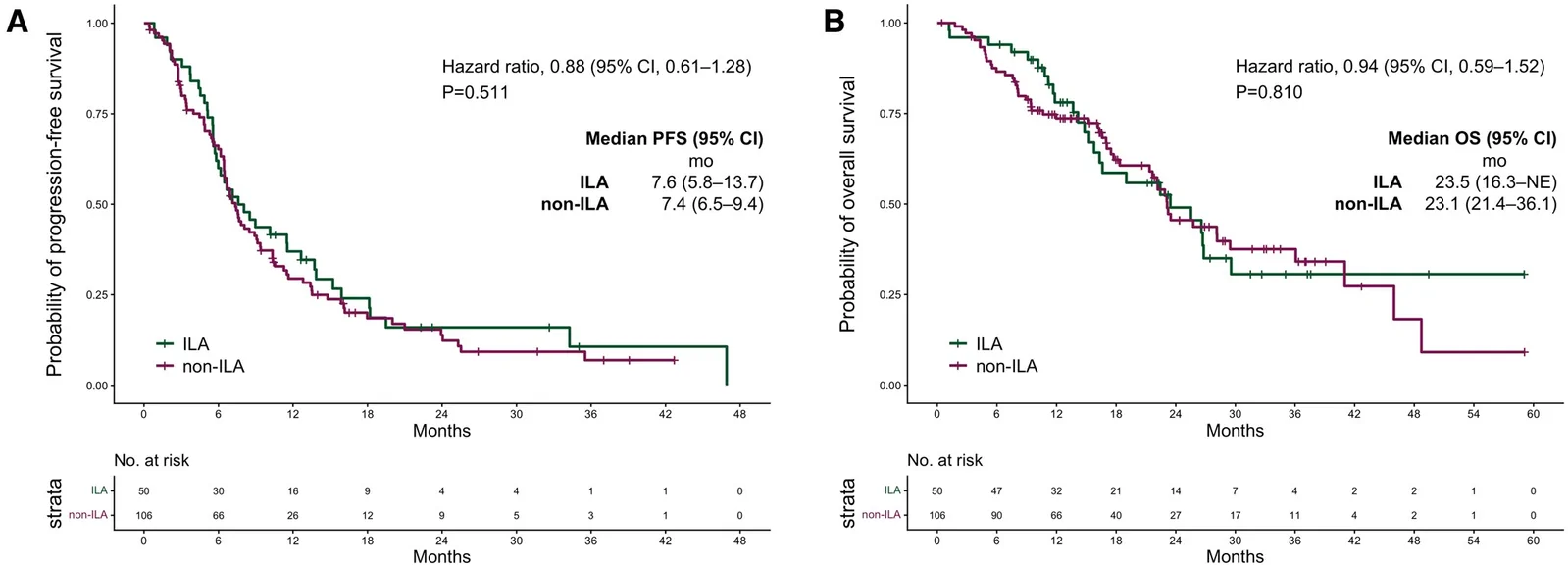
Progression-free and overall survival according to baseline ILA status. (A) Progression-free survival. (B) Overall survival. ILA, interstitial lung abnormality; NE, not estimable; T-DXd, trastuzumab deruxtecan.

### Timing of ILA detection before T-DXd initiation

Patient-level timelines of ILA detection before T-DXd treatment are shown in Figure 3D, with representative CT images shown in Figure 3A-C. Among the 50 patients with baseline ILA before T-DXd initiation, 17 had ILA already present before the start of systemic anticancer therapy, whereas 33 had ILA first detected during systemic anticancer treatment before T-DXd initiation. The median duration from initiation of systemic anticancer therapy for advanced or recurrent breast cancer to T-DXd initiation was 17.1 months (IQR, 3.8-30.8) in the pretreatment ILA group and 43.7 months (IQR, 25.1-70.9) in the treatment-course ILA group (*P* < .001). Baseline characteristics according to the timing of ILA detection are shown in Table S9.

**Figure 3.**
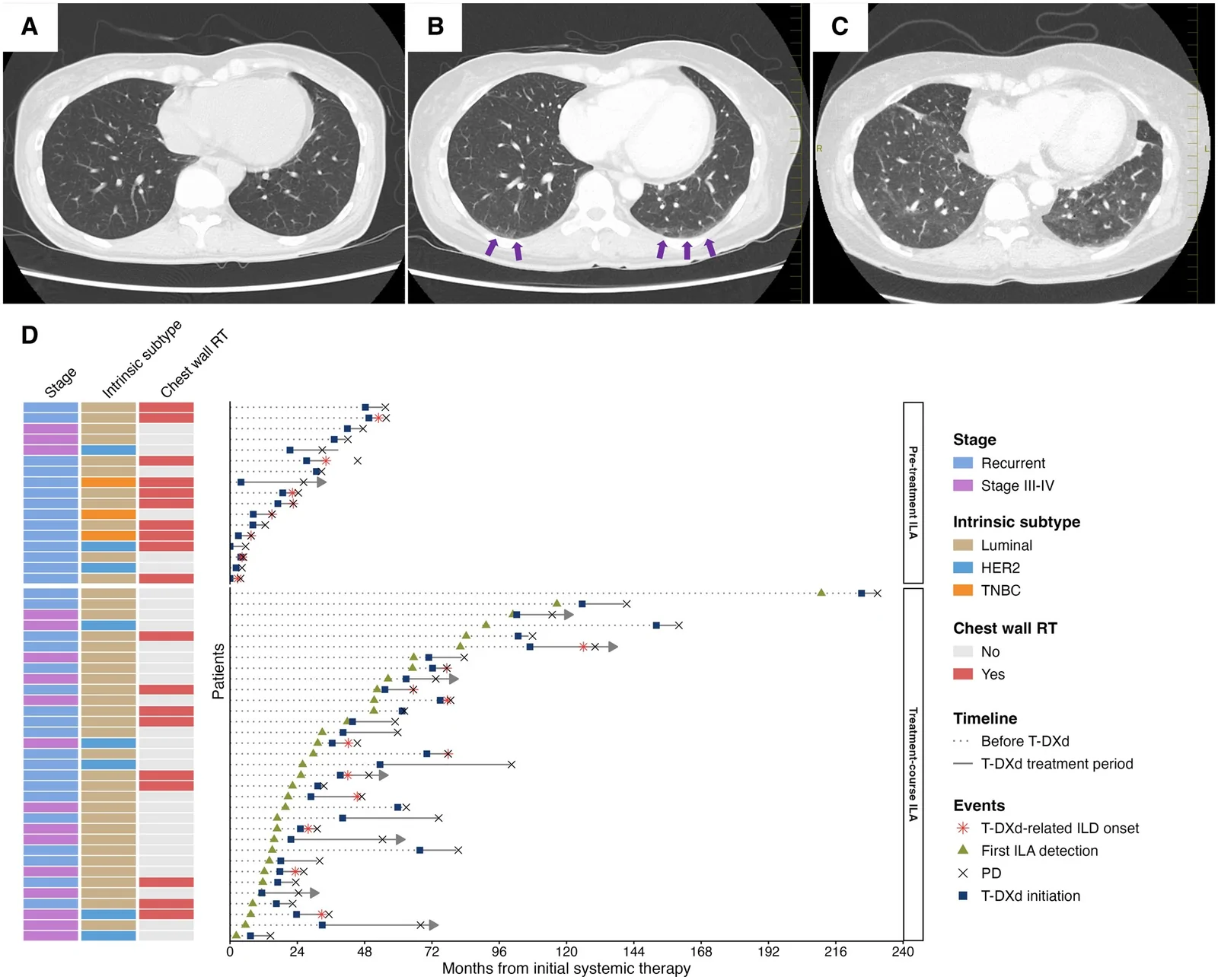
Representative CT images and patient-level timeline of ILA detection before T-DXd treatment. (A) Baseline CT before initial systemic anticancer therapy showing no ILA. (B) Follow-up CT during systemic anticancer therapy showing ILA first detected before T-DXd initiation, indicated by arrows. (C) Representative CT image of T-DXd-related ILD with a hypersensitivity pneumonitis pattern. (D) Patient-level timeline of ILA detection before T-DXd treatment. Time zero was defined as the date of initiation of systemic anticancer therapy for advanced or recurrent breast cancer. Patients in the pre-treatment ILA group had ILA already present before time zero, whereas patients in the treatment-course ILA group had no ILA before time zero and had ILA first detected during systemic anticancer therapy before T-DXd initiation. Arrows indicate ongoing T-DXd treatment. ILA, interstitial lung abnormality; ILD, interstitial lung disease; PD, progressive disease; RT, radiotherapy; T-DXd, trastuzumab deruxtecan; TNBC, triple-negative breast cancer.

## Discussion

In this retrospective cohort study of patients with advanced or recurrent breast cancer treated with T-DXd, baseline ILAs on CT before T-DXd initiation were associated with a higher incidence of T-DXd-related ILD. Importantly, equivocal ILA was associated with an increased risk of T-DXd-related ILD, and no significant difference was detected between the equivocal and definite ILA groups, although the CIs were wide. This association was also observed in the subgroup of patients evaluated with CT slice thickness <1.5 mm. These findings suggest that subtle interstitial abnormalities on baseline CT may be clinically relevant for predicting T-DXd-related pulmonary toxicity.

Previous pooled analyses of T-DXd monotherapy trials identified several risk factors for T-DXd-related ILD, including older age, Japanese origin, preexisting lung comorbidities, renal dysfunction, longer time since diagnosis, higher dose, and lower baseline oxygen saturation.^4^ However, “lung comorbidities” represent heterogeneous clinical entities and may not adequately capture subtle interstitial abnormalities on CT. More refined CT-based assessment may therefore be needed to identify patients with subclinical pulmonary vulnerability. In this context, accumulating evidence from other cancer treatment settings has shown that ILAs are associated with treatment-related pulmonary toxicity, particularly immune checkpoint inhibitor-related ILD and radiation pneumonitis.^6^^,^^7^^,^^10^ These observations support the clinical relevance of CT-defined ILAs for treatment-related pulmonary toxicity risk assessment. In breast cancer treated with T-DXd, a recent real-world cohort also reported pretreatment ILAs as a strong risk factor for pneumonitis.^11^ However, ILA assessment in that study was based on available clinical imaging, including PET-CT, and CT acquisition parameters were not explicitly incorporated into the analysis. In contrast, our study excluded patients with PET-CT only, used a structured ILA classification on baseline chest CT, distinguished equivocal from definite ILA, and explicitly evaluated CT slice thickness, including a sensitivity analysis restricted to patients with slice thickness <1.5 mm. Therefore, our findings support and refine previous observations by showing that CT-defined ILAs, including equivocal abnormalities, are associated with T-DXd-related ILD under more standardized imaging conditions.

A key finding of this study was that equivocal ILA was associated with an increased risk of T-DXd-related ILD, and no significant difference was detected between equivocal and definite ILA, although the CIs were wide. In our study, equivocal ILA referred to limited abnormalities that did not meet the conventional radiological threshold for definite ILA, including abnormalities involving <5% of any lung zone or unilateral abnormalities. Previous studies in lung cancer have suggested that equivocal or indeterminate ILAs are not necessarily benign, showing associations with shorter survival and acute exacerbation of interstitial pneumonia.^12^^,^^13^ However, the clinical impact of equivocal ILA on treatment-related ILD has not been well characterized. In the context of treatment-related ILD, Shimoji et al.^7^ reported that preexisting ILAs were associated with immune checkpoint inhibitor-related ILD in patients with non-lung cancers. Notably, their definition of ILA included limited abnormalities and did not separately evaluate equivocal and definite ILAs. Our study extends these observations by separately evaluating equivocal and definite ILA and showing that equivocal ILA was associated with an increased risk of T-DXd-related ILD.

The explicit evaluation of CT slice thickness is another strength of this study. Thin-section CT is more sensitive than thicker sections for detecting subtle ILAs, and current statements recommend thin-section imaging, generally <1.5 mm, for ILA assessment.^5^^,^^8^ In pivotal T-DXd trials, CT protocols were primarily designed for oncological assessment and generally allowed a slice thickness up to 5 mm, which may be adequate for tumor evaluation but less sensitive for subtle ILAs.^1–3^ In the present cohort, definite ILA was observed in 29 of 156 patients (18.6%), which was broadly consistent with the prevalence of ILAs reported in previous oncology cohorts.^7^^,^^14^ When equivocal ILA was included, however, the prevalence increased to 50 of 156 patients (32.1%). This higher prevalence likely reflects the inclusion of limited abnormalities below the conventional threshold for definite ILA and the detailed review of baseline CT images, most of which were obtained with a slice thickness <1.5 mm. However, the relatively permissive definition of equivocal ILA may have increased sensitivity at the expense of specificity, and some subtle findings may have been over-classified as equivocal ILA. In our cohort, the proportion of patients evaluated with CT slice thickness <1.5 mm was similar between the ILA and non-ILA groups. The association between baseline ILA and T-DXd-related ILD was also observed in the <1.5-mm CT subgroup.

Another notable finding was that ILAs were not always present before systemic anticancer therapy for advanced or recurrent breast cancer, but were frequently first detected during the treatment course before T-DXd initiation. However, the longer duration of systemic anticancer therapy before T-DXd initiation in the treatment-course ILA group (median, 43.7 vs 17.1 months) provided a greater opportunity for serial imaging and may have contributed to the timing of ILA detection. Longitudinal population-based studies have shown that ILAs can newly develop or progress over time.^15^^,^^16^ In oncology settings, evidence regarding the emergence or progression of ILAs during cancer treatment remains limited. However, radiologic progression of ILAs has been reported after lung cancer surgery, particularly among patients with baseline equivocal or definite ILA, and adjuvant thoracic radiotherapy has been identified as a factor associated with ILA progression in that setting.^17^ In advanced or recurrent breast cancer, patients may survive for prolonged periods and receive multiple lines of systemic anticancer therapy, and some also undergo radiotherapy or experience pulmonary infections or other inflammatory events during the disease course.^18–20^ Given this complex clinical trajectory and the retrospective design of this study, the causal relationship between these exposures and newly detected ILAs during the treatment course remains uncertain. Clinically, however, the key issue for T-DXd-related ILD risk assessment is whether ILA is present immediately before T-DXd initiation. Therefore, careful assessment of the most recent pre-T-DXd CT, preferably with thin-section images when available, is important for evaluating the risk of T-DXd-related ILD.

No significant differences in PFS, OS, or TTD were observed according to baseline ILA status in this cohort. However, the CIs for PFS and OS were wide and did not exclude clinically relevant effects. The lack of statistically significant differences in these outcomes may partly reflect the predominance of grade 1 or 2 T-DXd-related ILD events, the low frequency of grade ≥3 ILD, and the absence of recurrent ILD after T-DXd rechallenge in selected patients. In contrast, studies in lung cancer have reported associations of ILAs with worse survival and acute exacerbation of interstitial pneumonia.^12^^,^^13^ However, the reasons for these associations are not fully understood and may not be directly applicable to patients with breast cancer. Thus, the prognostic impact of baseline ILA on cancer outcomes remains uncertain, although baseline ILA remained clinically relevant for assessing the risk of T-DXd-related ILD.

This study has several limitations. First, this was a retrospective, single-center study with a limited number of ILD events, restricting multivariable adjustment and leaving potential for residual confounding. Predominance of Japanese women (153/156, 98.1%) limits generalizability, particularly given ethnic differences in T-DXd-related ILD risk. Second, CT protocols and imaging intervals were not standardized, while study-specific ILA categories were assigned retrospectively and did not guide surveillance. Despite substantial overall interobserver agreement, equivocal ILA agreement was limited (47.3%). Unblinded ILD adjudication could bias attribution of new opacities; together with numerically more frequent CT imaging in the ILA group, residual surveillance and ascertainment bias cannot be excluded. Third, the number of severe ILD events was small, and further studies are needed to evaluate the association between baseline ILA and severe T-DXd-related ILD.

## Conclusion

Baseline ILAs before T-DXd initiation were associated with an increased risk of T-DXd-related ILD in patients with advanced or recurrent breast cancer. No significant difference was detected between equivocal and definite ILA, although CIs were wide. Because ILAs may be first detected during prior systemic anticancer therapy even when absent at the start of treatment for advanced or recurrent disease, careful assessment of the most recent baseline CT may be important for risk assessment and monitoring before T-DXd initiation.

## Supplementary Material

oyag420_Supplementary_Data

## Data Availability

The data underlying this article will be shared on reasonable request to the corresponding author.
