# Supplementary material for: Equivocal and definite interstitial lung abnormalities as risk factors for trastuzumab deruxtecan-related interstitial lung disease in breast cancer
Source: Oncologist. 2026 Oct 19;31(11):oyag420. doi: 10.1093/oncolo/oyag420 (PMC13716483; doi:10.1093/oncolo/oyag420)
Supplement: Supplementary file 1 [file oyag420_supplementary_data.zip › Supplementary Table & Figure_clean_version.docx]

**Supplementary Table 1. Baseline characteristics of patients with baseline CT slice thickness <1.5 mm**

| **Characteristic** | **Total**  N = 123 | **ILA**  N1 = 40 | **non-ILA**  N2 = 83 |
| --- | --- | --- | --- |
| **Age, years** | 55.0 (49.0, 62.0) | 58.0 (53.0, 63.0) | 54.0 (45.0, 62.0) |
| **Sex** |  |  |  |
| Female | 123 (100%) | 40 (100%) | 83 (100%) |
| **Ethnic/geographic background** |  |  |  |
| Japanese | 120 (98%) | 38 (95%) | 82 (99%) |
| non-Japanese^a^ | 3 (2.4%) | 2 (5.0%) | 1 (1.2%) |
| **Smoking history** | 16 (13%) | 7 (18%) | 9 (11%) |
| **Performance status** |  |  |  |
| 0–1 | 108 (88%) | 37 (93%) | 71 (86%) |
| 2–4 | 15 (12%) | 3 (7.5%) | 12 (14%) |
| **Stage** |  |  |  |
| Recurrent | 84 (68%) | 26 (65%) | 58 (70%) |
| Stage III–IV | 39 (32%) | 14 (35%) | 25 (30%) |
| **Intrinsic subtype** |  |  |  |
| Luminal | 99 (80%) | 32 (80%) | 67 (81%) |
| HER2 | 20 (16%) | 6 (15%) | 14 (17%) |
| TNBC | 4 (3.3%) | 2 (5.0%) | 2 (2.4%) |
| **Liver metastasis** | 69 (56%) | 27 (68%) | 42 (51%) |
| **Brain metastasis** | 14 (11%) | 3 (7.5%) | 11 (13%) |
| **Creatinine clearance, mL/min^b^** | 86.9 (69.0, 104.6) | 87.0 (74.9, 107.4) | 86.9 (66.8, 102.9) |
| **Prior treatment** |  |  |  |
| Breast/chest wall RT | 48 (39%) | 16 (40%) | 32 (39%) |
| ICI | 6 (4.9%) | 1 (2.5%) | 5 (6.0%) |
| T-DM1 | 24 (20%) | 10 (25%) | 14 (17%) |
| **Number of prior treatment lines** | 3.0 (2.0, 5.0) | 2.5 (2.0, 5.5) | 3.0 (2.0, 4.0) |
| **Baseline CT slice thickness, mm** |  |  |  |
| 0.5 | 25 (20%) | 5 (13%) | 20 (24%) |
| 0.625 | 79 (64%) | 30 (75%) | 49 (59%) |
| 1 | 3 (2.4%) | 2 (5.0%) | 1 (1.2%) |
| 1.25 | 16 (13%) | 3 (7.5%) | 13 (16%) |
| **ILA type on baseline CT** |  |  |  |
| Equivocal ILA | — | 15 (38%) | — |
| Definite ILA | — | 25 (63%) | — |
| **Initial T-DXd dose, mg/kg** |  |  |  |
| 5.4 | 110 (89%) | 34 (85%) | 76 (92%) |
| 4.4 | 12 (9.8%) | 6 (15%) | 6 (7.2%) |
| 3.2 | 1 (0.8%) | 0 (0%) | 1 (1.2%) |

Continuous variables are expressed as medians (interquartile ranges), and categorical variables are expressed as n (%).

^a^ The three patients of non-Japanese background were Chinese, Malaysian, and Filipino (n = 1 each).

^b^ Creatinine clearance was estimated using the Cockcroft–Gault equation.

Abbreviations: ECOG PS, Eastern Cooperative Oncology Group performance status; ICI, immune checkpoint inhibitor; ILA, interstitial lung abnormality; RT, radiotherapy; T-DM1, trastuzumab emtansine; T-DXd, trastuzumab deruxtecan; TNBC, triple-negative breast cancer.

**Supplementary Table 2. Interobserver agreement for baseline ILA classification**

| **Group** | **N** | **Linear weighted kappa (95% CI)** | **Exact agreement, n (%)** | **Equivocal ILA-specific agreement, %** |
| --- | --- | --- | --- | --- |
| Overall | 156 | 0.721 (0.646–0.793) | 127 (81.4) | 47.3 |
| Prior RT: Yes | 62 | 0.693 (0.555–0.815) | 49 (79.0) | 23.5 |
| Prior RT: No | 94 | 0.739 (0.651–0.829) | 78 (83.0) | 57.9 |

Linear weighted Cohen's kappa was calculated using the independent ratings before consensus adjudication. The 95% confidence intervals were estimated by percentile bootstrap with 20,000 resamples. Exact agreement was defined as the proportion of patients assigned to the same ILA category by both readers. Equivocal ILA-specific agreement was calculated as twice the number of patients classified as equivocal ILA by both readers divided by the sum of patients classified as equivocal ILA by each reader.

Abbreviations: ILA, interstitial lung abnormality; RT, breast/chest wall radiotherapy; CI, confidence interval.

**Supplementary Table 2 (continued). Cross-tabulation of independent ratings**

|  |  | **Reader 2 classification** | | |  |
| --- | --- | --- | --- | --- | --- |
| **Group** | **Reader 1 classification** | **No ILA** | **Equivocal ILA** | **Definite ILA** | **Total** |
| Overall | No ILA | 105 | 5 | 0 | 110 |
| Overall | Equivocal ILA | 1 | 13 | 0 | 14 |
| Overall | Definite ILA | 0 | 23 | 9 | 32 |
| Overall | Total | 106 | 41 | 9 | 156 |
| Prior RT: Yes | No ILA | 42 | 3 | 0 | 45 |
| Prior RT: Yes | Equivocal ILA | 1 | 2 | 0 | 3 |
| Prior RT: Yes | Definite ILA | 0 | 9 | 5 | 14 |
| Prior RT: Yes | Total | 43 | 14 | 5 | 62 |
| Prior RT: No | No ILA | 63 | 2 | 0 | 65 |
| Prior RT: No | Equivocal ILA | 0 | 11 | 0 | 11 |
| Prior RT: No | Definite ILA | 0 | 14 | 4 | 18 |
| Prior RT: No | Total | 63 | 27 | 4 | 94 |

Rows represent Reader 1 classifications and columns represent Reader 2 classifications. All ratings were performed independently before consensus adjudication. RT, breast/chest wall radiotherapy.

**Supplementary Table 3. Fine–Gray and cause-specific Cox analyses of T-DXd-related ILD in the overall cohort.**

| **Model** | **Variable** | **Fine-Gray model: sHR (95% CI)** | **Fine-Gray p-value** | **Cause-specific Cox model: HR (95% CI)** | **Cause-specific p-value** |
| --- | --- | --- | --- | --- | --- |
| Univariable | ILA vs non-ILA | 3.74 (1.82–7.69) | <0.001 | 3.26 (1.58–6.73) | 0.001 |
| Multivariable | ILA vs non-ILA | 3.56 (1.63–7.78) | 0.001 | 3.18 (1.50–6.78) | 0.003 |
|  | Age, per 10-year increase | 1.11 (0.78–1.58) | 0.551 | 1.20 (0.81–1.78) | 0.352 |
|  | Breast/chest wall RT, Yes vs No | 1.38 (0.69–2.74) | 0.360 | 1.68 (0.81–3.48) | 0.165 |
| Sensitivity | ILA vs non-ILA | 3.53 (1.62-7.70) | 0.002 | 3.14 (1.48-6.69) | 0.003 |
|  | Age, per 10-year increase | 1.11 (0.78-1.58) | 0.566 | 1.20 (0.81-1.78) | 0.368 |
|  | Chest wall RT, Yes vs No | 1.35 (0.66-2.76) | 0.415 | 1.62 (0.77-3.42) | 0.207 |
|  | Smoking history, Yes vs No | 1.17 (0.47-2.93) | 0.730 | 1.24 (0.46-3.35) | 0.673 |

The multivariable model included ILA status, age, and prior breast/chest wall radiotherapy. The sensitivity model additionally included smoking history.

Abbreviations: CI, confidence interval; HR, hazard ratio; sHR, subdistribution hazard ratio; ILA, interstitial lung abnormality; **ILD, interstitial lung disease**; RT, radiotherapy; T-DXd, trastuzumab deruxtecan.

**Supplementary Table 4. Pairwise Gray tests for the cumulative incidence of T-DXd-related ILD according to baseline ILA classification.**

| **Comparison** | **Overall cohort Gray p-value** | **Overall cohort Holm-adjusted p-value** | **<1.5-mm subgroup Gray p-value** | **<1.5-mm subgroup Holm-adjusted p-value** |
| --- | --- | --- | --- | --- |
| Definite ILA vs non-ILA | 0.004 | 0.008 | 0.001 | 0.003 |
| Equivocal ILA vs non-ILA | <0.001 | 0.002 | <0.001 | <0.001 |
| Definite ILA vs Equivocal ILA | 0.638 | 0.638 | 0.537 | 0.537 |

Abbreviations: ILA, interstitial lung abnormality; **ILD, interstitial lung disease**; T-DXd, trastuzumab deruxtecan.

**Supplementary Table 5. Distribution of ILA subcategories and T-DXd-related ILD events among patients with definite ILA**

| **Definite ILA subtype** | **Patients, n** | **T-DXd-related ILD, n (%)** |
| --- | --- | --- |
| Non-subpleural | 7 | 5 (71.4%) |
| Subpleural non-fibrotic | 22 | 5 (22.7%) |
| Subpleural fibrotic | 0 | 0 |

Abbreviations: ILA, interstitial lung abnormality; **ILD, interstitial lung disease**; T-DXd, trastuzumab deruxtecan.

**Supplementary Table 6. Fine–Gray and cause-specific Cox analyses of T-DXd-related ILD in the <1.5-mm CT subgroup.**

| **Model** | **Variable** | **Fine-Gray model: sHR (95% CI)** | **Fine-Gray p-value** | **Cause-specific Cox model: HR (95% CI)** | **Cause-specific p-value** |
| --- | --- | --- | --- | --- | --- |
| Univariable | ILA vs non-ILA | 4.84 (2.18–10.77) | <0.001 | 4.16 (1.86–9.31) | <0.001 |
| Multivariable | ILA vs non-ILA | 4.53 (2.03–10.14) | <0.001 | 3.98 (1.75–9.04) | <0.001 |
|  | Age, per 10-year increase | 1.17 (0.79–1.74) | 0.429 | 1.20 (0.79–1.83) | 0.397 |
|  | Breast/chest wall RT, Yes vs No | 1.35 (0.65–2.81) | 0.420 | 1.62 (0.74–3.53) | 0.227 |

The multivariable model included ILA status, age, and prior breast/chest wall radiotherapy.

Abbreviations: CI, confidence interval; HR, hazard ratio; sHR, subdistribution hazard ratio; ILA, interstitial lung abnormality; **ILD, interstitial lung disease**; RT, radiotherapy; T-DXd, trastuzumab deruxtecan.

**Supplementary Table 7. Multivariable Cox analysis for PFS.**

| **Variable** | **HR (95% CI)** | **p-value** |
| --- | --- | --- |
| ILA vs non-ILA | 0.77 (0.51–1.15) | 0.202 |
| Age, per 10-year increase | 1.10 (0.92–1.31) | 0.317 |
| ECOG PS 2–4 vs 0–1 | 2.30 (1.36–3.88) | 0.002 |
| Stage III–IV vs recurrent | 0.99 (0.67–1.47) | 0.962 |
| Liver metastasis, Yes vs No | 1.80 (1.22–2.66) | 0.003 |
| Brain metastasis, Yes vs No | 1.32 (0.78–2.24) | 0.301 |
| Abbreviations: CI, confidence interval; ECOG PS, Eastern Cooperative Oncology Group performance status; HR, hazard ratio; ILA, interstitial lung abnormality; PFS, progression-free survival. | | |

**Supplementary Table 8. Multivariable Cox analysis for OS.**

| **Variable** | **HR (95% CI)** | **p-value** |
| --- | --- | --- |
| ILA vs non-ILA | 0.67 (0.40–1.12) | 0.125 |
| Age, per 10-year increase | 1.30 (1.02–1.65) | 0.033 |
| ECOG PS 2–4 vs 0–1 | 3.29 (1.74–6.20) | <0.001 |
| Stage III–IV vs recurrent | 1.44 (0.89–2.35) | 0.140 |
| Liver metastasis, Yes vs No | 2.88 (1.68–4.95) | <0.001 |
| Brain metastasis, Yes vs No | 1.16 (0.58–2.33) | 0.681 |
| Abbreviations: CI, confidence interval; ECOG PS, Eastern Cooperative Oncology Group performance status; HR, hazard ratio; ILA, interstitial lung abnormality; OS, overall survival. | | |

**Supplementary Table 9. Baseline characteristics of patients with ILA according to timing of ILA detection.**

| **Characteristic** | **Total**  N = 50 | **Pre-treatment ILA**  N1 = 17 | **Treatment-course ILA**  N2 = 33 | **p-value** |
| --- | --- | --- | --- | --- |
| **Age, years** | 60.5 (54.0, 66.0) | 62.0 (57.0, 69.0) | 60.0 (53.0, 63.0) | 0.260 |
| **Sex** |  |  |  |  |
| Female | 50 (100%) | 17 (100%) | 33 (100%) |  |
| **Smoking history** | 7 (14%) | 2 (12%) | 5 (15%) | >0.999 |
| **ECOG PS** |  |  |  | >0.999 |
| **0–1** | 45 (90%) | 15 (88%) | 30 (91%) |  |
| **2–4** | 5 (10%) | 2 (12%) | 3 (9.1%) |  |
| **Stage** |  |  |  | 0.080 |
| Recurrent | 33 (66%) | 14 (82%) | 19 (58%) |  |
| Stage III–IV | 17 (34%) | 3 (18%) | 14 (42%) |  |
| **Intrinsic subtype** |  |  |  | 0.050 |
| Luminal | 39 (78%) | 11 (65%) | 28 (85%) |  |
| HER2 | 8 (16%) | 3 (18%) | 5 (15%) |  |
| TNBC | 3 (6.0%) | 3 (18%) | 0 (0%) |  |
| **Liver metastasis** | 31 (62%) | 10 (59%) | 21 (64%) | 0.740 |
| **Brain metastasis** | 5 (10%) | 3 (18%) | 2 (6.1%) | 0.321 |
| **Prior treatment** |  |  |  |  |
| Breast/chest wall RT | 19 (38%) | 10 (59%) | 9 (27%) | 0.029 |
| ICI | 2 (4.0%) | 2 (12%) | 0 (0%) | 0.111 |
| T-DM1 | 14 (28%) | 4 (24%) | 10 (30%) | 0.746 |
| **Duration of systemic anticancer therapy before T-DXd initiation, months** | 34.7 (17.2, 61.0) | 17.1 (3.8, 30.8) | 43.7 (25.1, 70.9) | <0.001 |

Continuous variables are expressed as medians (interquartile ranges), and categorical data are expressed as n (%).

Abbreviations: ECOG PS, Eastern Cooperative Oncology Group performance status; ICI, immune checkpoint inhibitor; ILA, interstitial lung abnormality; RT, radiotherapy; T-DM1, trastuzumab emtansine; T-DXd, trastuzumab deruxtecan; TNBC, triple-negative breast cancer.

**Supplementary Figure 1. Patient flow diagram.**

ILA, interstitial lung abnormality; PET-CT, positron emission tomography-computed tomography; T-DXd, trastuzumab deruxtecan.

Alt text: Flow diagram showing patient selection and baseline ILA classification. Of 157 patients treated with T-DXd, one was excluded because baseline chest CT was unavailable. The remaining 156 patients were classified into the ILA group (n = 50; 21 equivocal and 29 definite ILA) and the non-ILA group (n = 106). T-DXd-related ILD occurred in 19 and 12 patients, respectively.

**Supplementary Figure 2. Cumulative incidence of T-DXd-related ILD in the <1.5-mm CT subgroup.**

**(A) Presence or absence of baseline ILA. (B) ILA classification. Between-group differences were assessed using Gray’s test.**

**ILD, interstitial lung disease; ILA, interstitial lung abnormality; T-DXd, trastuzumab deruxtecan.**

Alt text: Two panels showing cumulative incidence curves for T-DXd-related ILD among patients with baseline CT slice thickness below 1.5 mm. Panel A compares patients with baseline ILA (n = 40) and without ILA (n = 83), showing a higher cumulative incidence in the ILA group. Panel B compares no ILA, equivocal ILA (n = 15), and definite ILA (n = 25), showing higher cumulative incidences in both ILA categories than in the non-ILA group.
