# Supplementary material for: Equivocal and definite interstitial lung abnormalities as risk factors for trastuzumab deruxtecan-related interstitial lung disease in breast cancer
Source: Oncologist. 2026 Oct 19;31(11):oyag420. doi: 10.1093/oncolo/oyag420 (PMC13716483; doi:10.1093/oncolo/oyag420)
Supplement: Supplementary file 1 [file oyag420_supplementary_data.zip › Supplementary Figure1.pdf]

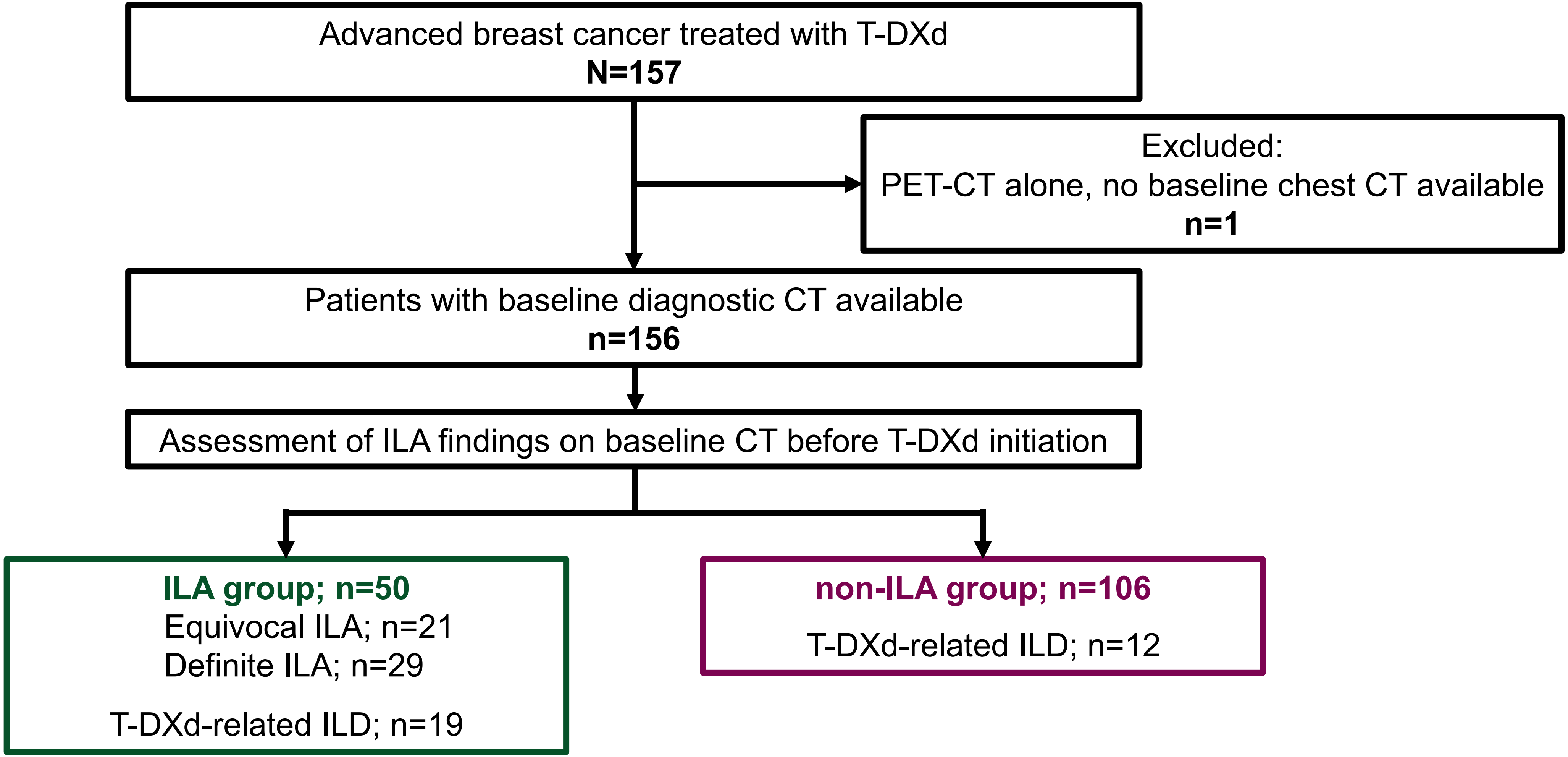

Advanced breast cancer treated with T-DXd
N=157
Excluded:
PET-CT alone, no baseline chest CT available
n=1
Patients with baseline diagnostic CT available
n=156
Assessment of ILA findings on baseline CT before T-DXd initiation
ILA group; n=50
Equivocal ILA; n=21
Definite ILA; n=29
T-DXd-related ILD; n=19
non-ILA group; n=106
T-DXd-related ILD; n=12
