# Supplementary material for: Equivocal and definite interstitial lung abnormalities as risk factors for trastuzumab deruxtecan-related interstitial lung disease in breast cancer
Source: Oncologist. 2026 Oct 19;31(11):oyag420. doi: 10.1093/oncolo/oyag420 (PMC13716483; doi:10.1093/oncolo/oyag420)
Supplement: Supplementary file 1 [file oyag420_supplementary_data.zip › Supplementary Figure2.pdf]

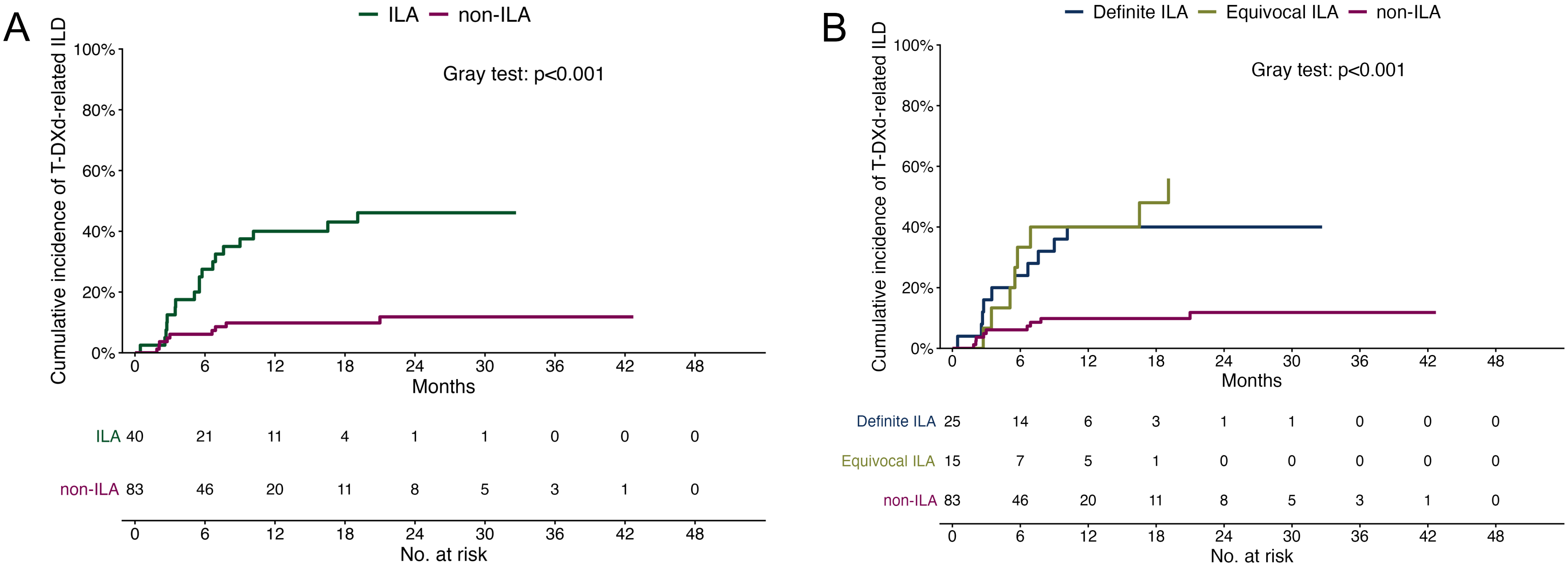
A
ILA
non-ILA
Cumulative incidence of T-DXd-related ILD
100%
80%
60%
40%
20%
0%
Gray test: p<0.001
0 6 12 18 24 30 36 42 48
Months
ILA 40 21 11 4 1 1 0 0 0
non-ILA 83 46 20 11 8 5 3 1 0
0 6 12 18 24 30 36 42 48
No. at risk
B
Definite ILA
Equivocal ILA
non-ILA
Cumulative incidence of T-DXd-related ILD
100%
80%
60%
40%
20%
0%
Gray test: p<0.001
0 6 12 18 24 30 36 42 48
Months
Definite ILA 25 14 6 3 1 1 0 0 0
Equivocal ILA 15 7 5 1 0 0 0 0 0
non-ILA 83 46 20 11 8 5 3 1 0
0 6 12 18 24 30 36 42 48
No. at risk
